# Implementing telemedicine for medical abortion within the public health system: a qualitative study on implementation bottlenecks and solutions in South Africa

**DOI:** 10.1186/s12889-025-24690-0

**Published:** 2025-10-09

**Authors:** Simone Storey, Amanda Cleeve, Margit Endler

**Affiliations:** 1 School of Public Health, University of Cape Town, Cape Town, South Africa; 2 Department of Global Public Health, Karolinska Institutet, Stockholm, Sweden; 3 Department of Women’s and Children’s Health, Karolinska Institutet, Stockholm, Sweden

**Keywords:** Telemedicine, Medical abortion, Public health, Access, Implementation bottlenecks, South Africa

## Abstract

**Background:**

Abortion in South Africa is legal, but there are still many barriers to access and high utilisation of the informal sector. Telemedicine for medical abortion is an alternative model that has been found to be a safe, effective, and acceptable option to increase access to abortion services. This study aimed to understand how key informants view telemedicine for medical abortion and how they view potential bottlenecks and solutions concerning implementation in the public sector of South Africa.

**Methods:**

We conducted interviews between February and March 2023 with 19 key informants across telemedicine and medical abortion provision, policy, and research. Through a qualitative design, we analysed the interviews using inductive content analysis. We used Baker et al.’s model of the implementation pathway to conceptualise and discuss the findings.

**Results:**

The findings showed that respondents perceived telemedicine as a valuable complement to in-clinic care to increase access to safe abortions. Respondents identified clinical concerns and logistical challenges as implementation bottlenecks and believed these could be overcome with innovative thinking and by drawing on existing resources. They suggested that research, leadership, collaboration, and policy alignment would help increase stakeholder willingness and capacity to build health system readiness. Across the implementation process, respondents viewed it necessary to consider users’ needs and adapt to contextual differences.

**Conclusions:**

This study identified telemedicine as a valuable model for increasing access to safe abortion services. Respondents outlined considerations and actionable steps to overcome implementation bottlenecks and guide the implementation of telemedicine for medical abortion in the public sector of South Africa and similar settings.

**Supplementary Information:**

The online version contains supplementary material available at 10.1186/s12889-025-24690-0.

## Background

Providing safe and comprehensive abortion care supports individuals’ sexual and reproductive rights and is key to reducing the burden of preventable morbidity and mortality from unsafe abortions [1]. Medical abortion is a safe, effective and highly acceptable abortion method using two medications, mifepristone and misoprostol, or using misoprostol alone [2]. The development and availability of medical abortion have led to an expansion of different service delivery models, including the provision of medical abortion fully or partly supported by telemedicine [3].

Telemedicine for medical abortion uses technology, such as telephone or email, to provide abortion care when there is a distance between the user and provider [4]. The evidence base has identified telemedicine as an effective and acceptable support for medical abortion in the first trimester with similar safety outcomes to in-clinic care, and the WHO now recommends its use [3, 5]. A telemedicine model for medical abortion typically includes some or all of the following components: eligibility screening, counselling, medication instructions, and follow-up care [3]. Several countries have implemented telemedicine for medical abortion and demonstrated its effectiveness, including Australia [6, 7], Mexico [8], the UK [9], and the US [10, 11]. Research also suggests that telemedicine can help to evade stigma and increase access to abortion care for underserved groups, such as people in rural areas and those relying on public services [8, 12].

In South Africa, abortion is legal up to 13 weeks of gestation on request; up to 21 weeks under certain conditions, including cases of rape and if the continued pregnancy would significantly affect the pregnant person’s social or economic circumstances; and after 21 weeks in specific cases of severe foetal abnormalities or when the pregnancy endangers the pregnant person’s life [13]. Despite the country’s liberal laws, many care seekers still face significant barriers to accessing abortion care, such as limited information on safe abortion services and unregulated belief-based denial of care by providers [14, 15]. Almost half (45%) of abortion seekers do not receive care on their first clinic visit, resulting in delayed or denied care [16]. Consequently, South Africa has a higher proportion of second-trimester abortions than other legal settings and higher usage of abortion methods outside the formal health sector, some of which may be unsafe and carry a higher risk of complications [17, 18].

In response to these barriers to access and the strict lockdowns against COVID-19, non-profit organisations began supporting medical abortions through telephone and email services [19]. Although the public sector has yet to implement telemedicine for medical abortion, organisations such as Marie Stopes South Africa and Abortion Support South Africa currently provide these services at varying levels of cost to users [20, 21]. Most South Africans rely on the public healthcare sector, which is state funded but frequently faces budget cuts, decreasing its capacity to meet the population’s health needs, especially in rural areas [22].

A randomised controlled trial (RCT) and sub-study found telemedicine for medical abortion to be effective and acceptable, and showed no indications of reduced safety in the South African context [23, 24]. Users, providers, and policymakers have shown high acceptance of this mode of service delivery, which can increase user self-efficacy and reduce the burden on healthcare facilities [24]. However, providers and policymakers have raised the need to address potential implementation challenges, such as improving information and communication technology (ICT) infrastructure and developing standard guidelines for telemedicine services [24]. Questions remain surrounding the feasibility of integrating and expanding telemedicine models within the South African public health sector, where it has yet to be implemented.

Implementation science offers theories, frameworks, and models to promote the adoption of evidence-based practices into routine healthcare services [25]. Studies examining the implementation of telemedicine for medical abortion in Colombia and the US identified components of successful implementation, including organisational readiness, motivated champions, and collaboration with innovators. They also highlighted regulatory barriers and providers’ limited ICT skills as obstacles [12, 26]. However, researchers have yet to explore these factors within the South African context. Addressing this lack of knowledge could accelerate implementation efforts, bridge the gap between evidence and practice, and expand abortion access with meaningful impacts on sexual and reproductive health and rights.

This study aimed to understand how key informants view telemedicine for medical abortion and how they view potential bottlenecks and solutions concerning implementation in the public sector of South Africa.

## Methods

### Study design and setting

We performed a qualitative study based on interviews with stakeholders involved in abortion care in South Africa. The study followed an RCT on telemedicine and abortion in the same setting [23] and was part of the evaluation of this intervention [24]. We employed an inductive qualitative approach to explore the perspectives of key informants. We defined key informants as experts with professional experience in telemedicine or medical abortion across the private, public, research, and civil society sectors. We conducted semi-structured interviews either in person in Cape Town or online. The study was reported according to the Consolidated Criteria for Reporting Qualitative Research (COREQ) [27].

Tanahashi’s model of health service coverage provides a foundation for measuring coverage of an intervention in the target population and identifying bottlenecks in implementation [28]. The implementation pathway, an adaptation of Tanahashi’s model by Baker et al., describes the stages of accessibility coverage, availability coverage, and effective coverage and shows where implementation bottlenecks can be identified between these stages [29]. We drew on these understandings of implementation bottlenecks to conceptualise the research questions, develop the interview guide, and interpret the results. Furthermore, we used Baker et al.’s model of the implementation pathway to contextualise our results and understand how these challenges impact the potential coverage of telemedicine for medical abortion in the South African public sector.

### Participant recruitment

We used purposive sampling, guided by a mapping of experts with specific experiences and roles in the provision of abortion care services, systems, and policies applicable to abortion care or telemedicine policy in South Africa (such as abortion providers, policymakers, ICT specialists, lawyers, or researchers). We also used snowball sampling, following recommendations from initial participants. We contacted 25 professionals via phone or email to explain the study’s purpose and invite their participation. Nineteen agreed to participate, while six did not respond. After confirming their interest, we sent participants a brief description of telemedicine for medical abortion along with additional resources for those unfamiliar with the topic. We determined the sample size was sufficient to address the research questions once we had interviewed a diverse range of professionals.

### Data collection

We developed and pilot-tested a semi-structured interview guide to ask participants about their views on telemedicine as a service delivery model for medical abortion in South Africa, what factors might be preventing this service from being available in the public sector, and the changes needed to make this possible (see Appendix). Participants were also asked to reflect on the public healthcare system’s readiness for implementation and to describe what a successful telemedicine model for medical abortion might look like in the South African public sector.

SS conducted interviews during February and March 2023, either in person or online via Zoom or Microsoft Teams. Before each interview, we obtained written informed consent electronically or in person to take notes, audio-record, and transcribe the conversation verbatim. Interviews were conducted in English and lasted between 50 and 80 min. Participants were not compensated as they were invited to participate in their professional capacity. To protect confidentiality, we removed all identifying information during transcription. This study received ethical approval from the University of Cape Town Health Sciences Human Research Ethics Committee (HREC Ref: 837/2020) as an amendment to the ethical approval for the original RCT.

### Data analysis

We analysed interview transcripts in Dedoose [30] using inductive content analysis, a commonly used qualitative methodology to inform guidelines and policy development [31]. SS conducted the initial coding by identifying meaning units and inductively recording them as fine-grained codes through a predominantly manifest coding approach. The study team collaboratively organised the collection of codes and revised them into categories and subcategories to create a coding schema. We reread the data excerpts within each category and subcategory and discussed their interpretations through an iterative process. Baker et al.’s model of the implementation pathway and the research questions guided this analytical process [29]. We reflected on our perspectives and research interests through the process and endeavoured to limit the influence of personal bias during analysis and reporting. We included quotes to increase data dependability and the trustworthiness of the findings. While all participants’ perspectives are represented in the results, we selected appropriate quotes from some participants.

## Results

This study included 19 key informants, representing diverse backgrounds in abortion policy, research, and provision, as well as expertise in telemedicine, ICT infrastructure, and healthcare innovation. Thirteen participants were medically trained as doctors, midwives, or nurses, although many were currently working in a government department or research institution. Nine of the professionals worked at the national level, while the others brought experience from four different provinces. Additional participant characteristics are presented in Table 1.

**Table 1:** Participant characteristics

Participant characteristics	Number of participants
Role	
- Researcher	5
- Public provider	2
- Non-profit provider	2
- Private provider	2
- Provincial policymaker	2
- Public health ICT specialist	2
- Public health telemedicine specialist	1
- Public health education specialist	1
- Pharmaceutical expert	1
- Attorney	1
Sector	
- Public	8
- Non-profit	5
- Private	3
- Academic	3
Telemedicine experience	
- Provision	5
- Policy	5
- Research	2
- Infrastructure	2
- Limited/none	5

Our analysis generated four categories: (1) Telemedicine as a complement to in-clinic care: increasing access, options and autonomy; (2) Out-of-the-box thinking to overcome implementation bottlenecks; (3) Increasing willingness and capacity to build health system readiness; and (4) Not one size fits all: adapting telemedicine models to users and their contexts. The findings are presented in Table 2 according to categories.

**Table 2:** Categories and subcategories generated from inductive content analysis

1 Telemedicine as a complement to in-clinic care: increasing access, options and autonomy	
1.1 Telemedicine is easier and more accessible for users	1.2 In-clinic care is difficult but still important
2 Out-of-the-box thinking to overcome implementation bottlenecks	
2.1 Navigating clinical concerns with screening, information, and trust	2.2 Drawing on existing resources to address logistical challenges
3 Increasing willingness and capacity to build health system readiness	
3.1 Offsetting the resistance to change with leadership and research	3.2 Aligning partnerships and policies to build implementation capacity
4 Not one size fits all: adapting telemedicine models to users and their contexts	
4.1 Accommodating users’ contexts, needs, and preferences	4.2 Adapting to contextual differences and varying implementation readiness

Respondents perceived telemedicine as a valuable complement to in-clinic care with the potential to increase access to medical abortion. They identified various clinical and logistical bottlenecks to the implementation of telemedicine for medical abortion but also described ways to overcome these challenges, such as applying innovative thinking and utilising existing resources. To build health system readiness, respondents suggested increasing willingness with research and supportive leadership and building implementation capacity through strategic partnerships and policy alignment. They considered the bottlenecks to implementing telemedicine for medical abortion in the public sector as surmountable but emphasised the perceived need to consider contextual differences and provide a spectrum of telemedicine service models with responsive adaptations.

### 1 Telemedicine as a complement to in-clinic care: increasing access, options and autonomy

Although respondents described various challenges to accessing in-clinic care, they still viewed in-clinic care as necessary. They viewed telemedicine as valuable but positioned it as a complement to in-clinic care for increasing access.

#### 1.1 Telemedicine is easier and more accessible for users

Respondents relayed how telemedicine could increase access to abortion services by mitigating various barriers to in-clinic abortion care, such as insufficient abortion providers and belief-based denial of service. They described telemedicine as a potential channel to circumvent stigma and direct users to accessible services and supportive abortion providers. Respondents saw this as especially important for geographically dispersed communities where access to in-clinic services for abortion is limited.

*“I think it is an excellent opportunity to increase access*,* especially where we have health systems that are really constrained*,* where we have people who can’t always get to a health facility*,* where we have a lot of stigma around abortion services… It’s definitely something that can address so many limitations and barriers that we see today in reproductive health care.” (Researcher)*.

Respondents reasoned that telemedicine could decrease delays in accessing abortion care, thereby reducing the number of second-trimester abortions with higher costs and risks of complications. They further recounted benefits such as reducing the psychological capital of navigating in-clinic abortion services and alleviating experiences of judgment. Respondents perceived telemedicine as giving users a greater sense of autonomy and described it as less disruptive to individuals’ education, economic activity, and general health.

*“It would save her so much time. It would save her so much money. It would save her so much of the trauma of having to find shelter in a city far away from where you live until someone can help you with your situation.” (Attorney)*.

#### 1.2 In-clinic care is difficult but still important

Interviews revealed that respondents perceived several current challenges to accessing in-clinic care in South Africa, including overburdened healthcare facilities struggling to meet the high demand for abortion services, long travel times, inconvenient clinic opening hours, and users being denied access or turned away by gatekeepers at facilities.

*“They travel to the nearest city to try and get an abortion. They get there most of the time*,* and there’s a long line*,* and they have to then either sleep outside or*,* like*,* you know*,* just find some shelter until they can be seen… The demand is so much and the actual provision of the service is so low that even with telemedicine*,* there will still be a gap.” (Attorney)*.

Respondents viewed limited public abortion provision and unaffordable private services as drivers of unsafe abortion. While respondents agreed that telemedicine could decrease this gap in service delivery, they stressed that there will always be a need for in-clinic care, and telemedicine should operate in parallel to it rather than replacing it. Respondents highlighted the perceived importance of providing options for abortion care to meet users’ needs. They also emphasised the perceived need to build capacity and increase access to in-clinic services to meet the needs of users who are not eligible for telemedicine, prefer in-clinic care, or require treatment of post-abortion complications.

*“It’s a very good way of overcoming barriers to access. We know that many women prefer this option even if there are other options available. So it’s not just about improving access*,* it’s also about women’s choices and preferences. But that being said*,* we will always need brick-and-mortar clinics. It’s not an alternative. It’s additional.” (Non-profit provider)*.

### 2 Out-of-the-box thinking to overcome implementation bottlenecks

Respondents highlighted various perceived clinical and logistical bottlenecks to the implementation of telemedicine for medical abortion, but they usually presented solutions concurrently.

#### 2.1 Navigating clinical concerns with screening, information, and trust

Some respondents were apprehensive about the absence of an in-clinic visit and viewed this as a lost opportunity for additional health checks. A few raised concerns about the lack of ultrasound or physical examination to confirm gestational age and discover ectopic pregnancies. Some respondents were also worried that users would not accurately identify their gestational age (due to poor menstrual literacy or lying out of desperation) or that users would not administer the medication correctly.

*“You’d need to have quite a rigorous screening for the women that want to take part in telemedicine*,* and think if there’s any question mark on the validity or the accuracy or the honesty of the woman’s answers*,* then she needs to be referred for in-person care.” (Non-profit provider)*.

Respondents explained that these concerns could be mitigated by conducting thorough eligibility screening and giving clear instructions on how to take the medication, what to expect, how to determine success, and what to do in case of complications. They also suggested standard operating procedures, consent forms, clear referral pathways to in-clinic care, record-keeping for telemedicine consultations, and clarity on where responsibility lies.

*“In this case*,* we’re saving the woman’s life because the risk is that she will go to an illegal provider.” (Pharmaceutical expert)*.

Some respondents were worried that a telemedicine service would be used to get pills to sell informally and fuel unsafe abortions, while other respondents suggested telemedicine could shift more users to formal healthcare and reduce unsafe abortions. Respondents described increased comprehensive sexuality education, knowledge of reproductive rights, and awareness of where to access services as ways to help users identify their pregnancy earlier and know where to seek safe abortion care. Furthermore, some respondents expressed the perceived need to be patient, trust users, and rely on their agency and self-knowledge.

#### 2.2 Drawing on existing resources to address logistical challenges

##### Medication collection should be easy and have multiple options

Respondents noted that the combination of mifepristone and misoprostol is more effective but limited by the high cost of mifepristone. To address this, they suggested producing a generic version of mifepristone through a government tender. Respondents shared various ideas for how users could get abortion medications, such as couriers, decentralised pick-up locations, vending machines, or pharmacies. They referred to other distribution mechanisms for inspiration, like decentralised collection points for chronic medication and government partnerships with private pharmacies to provide contraceptives. While some respondents mentioned user verification during the collection or distribution of the medication, they emphasised that the focus should be on making the experience simple and safe for users.

*“In terms of collecting the medication*,* how can we make it easy for you to collect it? And because it might also not be possible to do a courier service in an informal settlement or something like that. So we need to think a little bit outside of the box and don’t think that it won’t work.” (Non-profit provider)*.

##### There are implementation costs, but telemedicine may be more cost-effective

Respondents discussed various telemedicine service models with differing resource needs, ranging from low-budget implementations to high-level structural costs. They speculated potential costs, including medications, call centres, and staff training. Several respondents raised concerns about the decreasing health budget due to competing demands and advised strategic partnerships with private organisations to manage resources. Regardless of the model, respondents emphasised that services should be free for users, with the economic burden on the government. Respondents described how the option of telemedicine could reduce costs by decreasing in-clinic costs, surgical abortion rates, and complications from unsafe abortions. While respondents generally expected telemedicine to be more cost-effective over time, some called for an economic evaluation to show the return on investment of implementing telemedicine.

*“But from cost-effectiveness or cost*,* like an economic evaluation side of things*,* we need to also think of the costs averted by implementing telehealth services… So with each call you make*,* there’s one less person in the clinic.” (Provincial policymaker)*.

##### Telemedicine can work now, but ICT infrastructure needs to be developed for scale-up

Some respondents believed the basic ICT infrastructure for implementation was already in place and recommended designing telemedicine models based on what already exists. Others argued that more advanced digital structures were needed, including an electronic registry with user information, data security management, ICT infrastructure in public facilities, as well as database linkages to share clinical management and include telemedicine users in caseload totals. To meet consent requirements, several respondents positioned verbal consent and recorded calls as easier and more practical than requiring a signed consent form. Respondents shared how telemedicine services are already happening to some degree on WhatsApp, but they raised concerns around data protection and privacy. Many respondents emphasised the perceived need for an official, secure digital platform for telemedicine.

*“At its most simplistic level*,* this could be implemented as a service on a proof of concept basis*,* if you like*,* using existing infrastructure and technologies and services. But if you really wanted to scale services more widely*,* then you need to get the dependencies in place.” (Public health ICT specialist)*.

##### Health workers’ roles can adapt to include telemedicine

Respondents shared different ideas of who would provide telemedicine services, who would be responsible for it, and whether it would be managed from a facility, provincial, or national level. Most respondents suggested that nurses trained in abortion provision could adopt telemedicine services with a doctor on call for queries. Others proposed involving trained personnel, like community healthcare workers and pharmacists, to play key roles in service delivery and follow-up. Respondents discussed adapting existing health worker roles to include telemedicine or creating new roles to exclusively deliver telemedicine services. Regardless of the model, they emphasised that providing telemedicine services should not increase the current burden on abortion providers. To facilitate sustainable implementation, respondents suggested engaging health workers in telemedicine service delivery and integration, as well as developing their ICT skills and professional confidence in the telemedicine system.

*“I think you develop a digital bedside manner when you do telemedicine… you have to have a specific kind of mindset to do this work.” (Public health telemedicine specialist)*.

### 3 Increasing willingness and capacity to build health system readiness

Respondents suggested that supportive leadership and advocacy from researchers could increase willingness to implement telemedicine. They also recommended forming strategic partnerships, updating training curricula, and aligning relevant policies to build implementation capacity.

#### 3.1 Offsetting the resistance to change with leadership and research

While respondents perceived an increased appreciation of telemedicine since the COVID-19 pandemic, many still sensed a lack of willingness to implement this within the public sector. They attributed this reluctance to fear of the unknown, resistance to change, and the sector’s high bar for clinical safety. To foster leadership support for telemedicine, respondents suggested actively engaging stakeholders, recognising champions, and showcasing innovative practices. They also framed advocacy and commitment from national and provincial leaders as facilitators of implementation and saw apathetic or anti-choice attitudes of people in power as barriers.

*“It’s difficult to change culture in a hospital if it isn’t a decree from above. There’s always fear… I think you would need to have a formal directive to say this is acceptable and we can do this.” (Public provider)*.

Respondents proposed allowing stakeholders to voice their concerns, acknowledging their apprehension, and using research to address these issues. They focused on sharing evidence of safety and examples of successful implementation to obtain buy-in. Furthermore, respondents called on researchers to become advocates and negotiate with the government to develop policy changes and build implementation capacity. They also encouraged researchers to share findings widely, engage with local leadership, and gain public support.

*“Researchers really hold the tools and the knowledge that can inform a successful intervention in our country… I think when we bring that together and we present it to the decision makers together*,* collectively*,* we solidify this approach that this is effective.” (Researcher)*.

#### 3.2 Aligning partnerships and policies to build implementation capacity

Some respondents believed that sufficient evidence and willingness already exist, but the government remains uncertain about how to move forward with implementing telemedicine services. They recommended piloting different models integrated with existing models of care to figure out what works, what needs to be improved, and what additional resources are needed. Respondents proposed partnerships across public, private, academic, and civil society sectors to support a collaborative model and suggested engaging with experts on telemedicine for medical abortion to guide sustainable implementation.

*“Government wants to implement it. I think they just don’t quite know how to do it… They need to rely on experts in this*,* and we can tell them this is safe. This is the international standard now*,* and the WHO says it’s safe.” (Non-profit provider)*.

To build implementation capacity and confidence, a few respondents recommended interdepartmental collaboration to update curricula for training healthcare professionals with the integration of telemedicine and new demands in practice. In addition to training providers on telemedicine procedures, some respondents highlighted training for other healthcare professionals on the option of telemedicine for medical abortion to assist with referral and reduce stigma. They also described the perceived importance of aligning policies, such as national telehealth guidelines and clinic designation processes, to integrate telemedicine for medical abortion.

*“It’s the responsibility of the service staff to consider how telehealth might make services to citizens better. Then it’s the responsibility of the policy and the system*,* i.e. us*,* to provide them with the tools*,* support*,* and guidelines they need to do that easily and safely.” (Provincial policymaker)*.

### 4 Not one size fits all: adapting telemedicine models to users and their contexts

Respondents emphasised the perceived value of contextually adapted and user-centred approaches to support the usability and acceptability of telemedicine for medical abortion.

#### 4.1 Accommodating users’ contexts, needs, and preferences

Respondents perceived that the usability of telemedicine for medical abortion would depend on users’ access to the internet, privacy, and language. Despite high digital penetration in South Africa, respondents noted that data was costly and suggested a data-free platform and toll-free call centre. While telemedicine could provide privacy for some users, some respondents discussed challenges for users who share a phone or live in confined spaces. To address this, they proposed a private space in facilities for users to utilise telemedicine services. Most respondents also recommended that the service should be designed to accommodate language barriers, such as using illustrations and confirming that the user understands the information.

*“Considering everybody and their abilities to access the service and to understand how to do it is important for a model in South Africa.” (Researcher)*.

Respondents expressed differing expectations about how acceptable telemedicine would be to users, with mixed views on whether it would be more popular for younger or older users. They anticipated that some users might initially lack trust in the service and recommended clear information to mitigate fears. Some respondents also suggested raising awareness through media campaigns and providing accessible information on abortion options to increase acceptability. Respondents elaborated that telemedicine models should be informed by users’ contextual challenges and responsive to their needs and preferences. They agreed that telemedicine would not be the right solution for everyone, but it could be a valuable option for many.

*“This isn’t going to be a one-size-fits-all for every single client. There are going to be some clients where telemedicine is appropriate and some clients where it’s not.” (Non-profit provider)*.

#### 4.2 Adapting to contextual differences and varying implementation readiness

Respondents reflected on the cultural, geographical, and socioeconomic diversity of South Africa, considering the value of telemedicine in areas with limited abortion services as well as the challenges to implementation in these contexts. A few respondents suggested targeting different community groups according to their needs and providing options for in-clinic care or connectivity for telemedicine services. Some also described marked provincial differences in abortion service provision and varying levels of readiness to adopt telemedicine as potential challenges to scaling up national telemedicine services. To navigate these differences, they advised engaging with provincial stakeholders to appropriately adapt telemedicine models.

*“We have a vastly varied population… with vastly different challenges and opportunities in those different areas. And so I think we need to acknowledge that we can’t have a provincial level like one size fits all.” (Provincial policymaker)*.

Respondents generally believed that implementation was possible but would require work and out-of-the-box thinking. Rather than calling for major organisational changes, most respondents emphasised the perceived need to figure out where telemedicine could fit within existing systems and to adapt service models accordingly.

*“Everything that you could consider to be a barrier or a risk is either already addressed in the research or is simply just a design barrier… just because something is difficult*,* doesn’t mean you can’t do it. You just have to address it.” (Provincial policymaker)*.

**Fig. 1:**
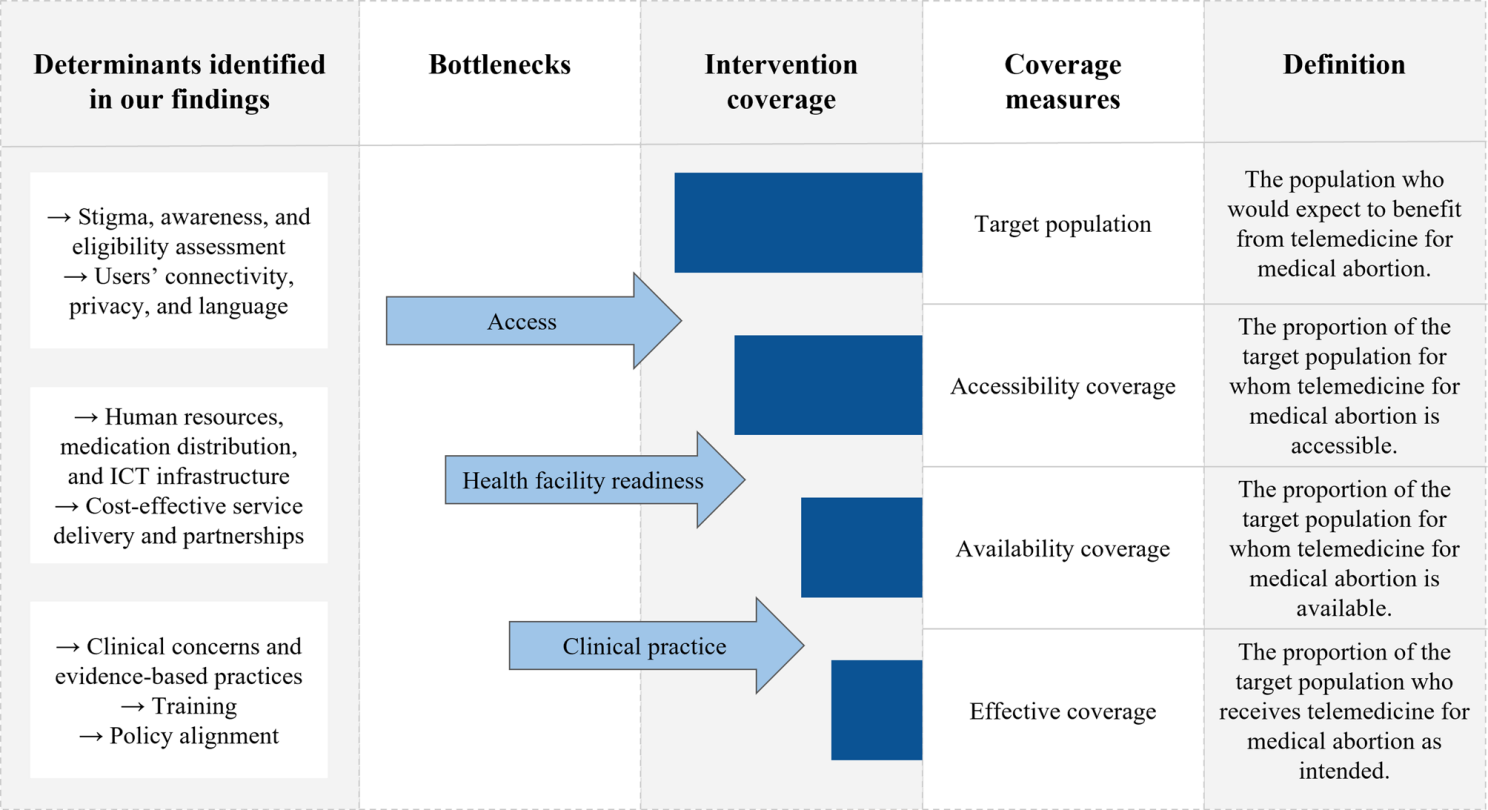
Findings in relation to Baker et al.’s implementation pathway

These findings mapped various bottlenecks to coverage of telemedicine for medical abortion along Baker et al.’s implementation pathway, as illustrated in Fig. 1.

## Discussion

Our results indicate that respondents perceived telemedicine for medical abortion as a valuable complement to in-clinic care but also identified various implementation bottlenecks and potential solutions. We examined our findings through the lens of Baker et al.’s model of the implementation pathway to understand what bottlenecks limit accessibility, availability, and effective coverage of telemedicine for medical abortion [29].

From the view of the implementation pathway, our study found that respondents positioned telemedicine for medical abortion as a beneficial intervention to increase coverage of safe abortion services in the public sector of South Africa. This mirrors previous findings from South Africa and other contexts that accessing in-clinic care can be difficult, and telemedicine for medical abortion could provide an easier and highly acceptable option for users and providers [9, 24, 32]. While respondents recognised the perceived merit of telemedicine, they also acknowledged that in-clinic care needed to be strengthened to support users who required or preferred in-clinic care.

According to Baker et al.’s implementation pathway, our findings revealed bottlenecks in access, which affect accessibility coverage, restricting the proportion of the target population for whom telemedicine for medical abortion would be accessible [29]. A scoping review on abortion stigma highlighted how stigma limits awareness of abortion rights, access to information, and referral to safe services, including telemedical services [33]. While our respondents described telemedicine as a channel to alleviate stigma, they also showed concern that users would struggle to correctly determine their gestational age to self-assess their eligibility for telemedicine services. Similar concerns were reported in Colombia, where poor menstrual knowledge was viewed as a barrier to accessing telemedicine for medical abortion [12]. To increase knowledge and acceptability, our respondents proposed improved comprehensive sexuality education and awareness campaigns, inspired by the positive impact of HIV awareness campaigns [34].

Respondents also perceived contextual differences in users’ connectivity, privacy, and language needs as potential barriers to access. A study on ethical considerations for mobile phone interventions in South Africa described how participants often shared phones and received messages for others, changed phone numbers due to theft, and struggled to afford airtime, get network coverage, or charge their phones [35]. Our respondents mirrored these concerns and suggested data- or toll-free options. Similarly, research in Mexico found that targeted telemedicine approaches better support underserved, geographically dispersed populations [8]. Our respondents also raised concerns about differences in language and literacy as implementation challenges and called for telemedicine models to accommodate language barriers and use visual aids. In addition, a study in the US on language-specific challenges to general telemedicine services recommended recruiting telemedicine providers who speak users’ languages and incorporating interpretation services into service delivery [36].

Baker et al.’s implementation pathway illustrates how bottlenecks in health facility readiness curb availability coverage, restricting the proportion for whom telemedicine for medical abortion is available [29]. Our results showed that the availability of telemedicine services depended on the type of delivery model, and respondents suggested working with existing systems to maximise implementation capacity, such as utilising available human resources, medication systems, and ICT infrastructure. Respondents encouraged using current abortion providers and addressing staffing constraints. Previous research supports this view, showing that a lack of trained providers hinders the integration of telemedicine and emphasising the importance of building organisational capacity before implementation by confirming the availability of trained providers [12, 37]. Our respondents’ perceptions of medication distribution aligned with a scoping review of medication distribution, which found that new methods, such as automated pharmacy dispensing units and smart lockers, offered cheaper and better alternatives for users than collecting medication from facilities [38]. A study in the US noticed that clinics with the required medication and ICT resources to provide telemedicine for medical abortion implemented services more efficiently [26]. Some of our respondents believed the required ICT infrastructure for telemedicine already existed, while others insisted on developing more complex digital systems.

Research on telemedicine for general health services in African settings reflects that limitations in ICT infrastructure can reduce the availability of services, but simple telemedicine technologies could optimise initial set-up costs [37, 39]. A review of telemedicine services in the OECD supports the notion of cost-effectiveness, although poor reporting and quality limited generalisability [40]. Similarly, our respondents perceived that telemedicine could be more cost-effective in the long term, but they encouraged an economic evaluation to determine this and inform multisectoral collaboration. Many respondents also highlighted public-private partnerships and collaboration with civil society organisations as central to building implementation capacity and health system readiness. In other contexts, research found that collaboration can support implementation feasibility, such as developing network infrastructure with mobile technology operators, receiving information materials from civil society organisations, and reaching agreements with delivery companies [12, 26, 41]. Our findings echo the perception that sustainable implementation depends on support from organisational leadership, champion providers, and engaged academics [26, 39, 41].

At the last stage of Baker et al.’s implementation pathway, bottlenecks in clinical practice influence effectiveness coverage, further impacting the proportion of the target population who receive telemedicine for medical abortion services as intended [29]. Our results identified clinical concerns about telemedicine for medical abortion regarding the lack of ultrasound or physical examination, despite evidence that the absence of ultrasound in telemedicine models does not result in more complications than models using ultrasound before an abortion and ultrasound dating could suitably be excluded for the majority of pregnant people who are certain of their last menstrual period [11, 42]. Our respondents also raised concerns about complications and misuse, which researchers have linked to the lower acceptability of implementing telemedicine for medical abortion among less experienced providers in Colombia [12]. Addressing these concerns, the RCT in South Africa found high adherence to medication instructions in addition to acceptability, effectiveness, and no indications of reduced safety [23]. Our findings supported promoting research dissemination to address knowledge gaps and encourage evidence-based practices, as reflected in an implementation study in the US [26].

Our respondents called for ICT training for telemedicine providers and increased awareness of telemedicine for medical abortion among other healthcare professionals to facilitate decision-making support and referral. Other implementation studies support the need for telehealth training, value clarification activities, and clear guidelines [12, 26]. Our respondents discussed integrating information on telemedicine for medical abortion and basic abortion procedures into the training curriculum for nurses. This connects to other research that calls for including abortion training in South African medical education as well as introducing general telemedicine technology [39, 43].

In South Africa and Colombia, implementation research has identified how policies can create barriers for those most in need of telemedicine services, like requirements for video calls, and can limit provision, such as strictly regulating telehealth in providers’ scope of practice [12, 41]. To support the provision of appropriate and effective telemedicine services, our findings indicated that policymakers would need to align policies accordingly. Our respondents also acknowledged that implementers should adapt service-delivery models according to varying access needs, implementation readiness, and clinical skills to increase the intervention coverage of telemedicine for medical abortion.

The findings of this study highlight key steps towards effective implementation: policymakers should align telehealth and medical abortion policies for consistency, implementers should map medication distribution systems to ensure telemedicine models achieve national coverage, relevant authorities should evaluate the national ICT infrastructure for practical integration with telemedicine, advocacy groups should conduct awareness campaigns to guide users and referrals toward available telemedicine services, and stakeholders should engage in establishing partnerships that expand access to telemedicine for medical abortion.

### Strengths and limitations

The application of a theoretical model strengthens the credibility of the results and informs the analysis of the identified bottlenecks to implementing telemedicine for medical abortion in South Africa and similar settings. By drawing on experiences from a wide range of experts across different sectors in South Africa, this study provided rich detail on perceived implementation bottlenecks and potential solutions. However, we selected key informants based on their connection to telemedicine or medical abortion, so the sample likely included more permissive individuals than the general population of healthcare professionals or associated professionals. This might have given a more positive impression of stakeholder readiness and the ability to overcome implementation bottlenecks than what exists in reality. Most informants also came from a management, policy, or research background, which limited the perspectives from potential public providers of telemedicine for medical abortion and stakeholders working on the ground.

## Conclusions

This study has shown that professionals perceived telemedicine as a valuable option for abortion provision that will increase access and convenience for users, especially in tandem with in-clinic care. However, they identified clinical concerns and logistical challenges as bottlenecks to implementation that stakeholders could overcome with innovative thinking and working with existing resources. Professionals also suggested building implementation readiness and adapting telemedicine models to contextual differences. Policymakers and providers should take active steps toward implementing telemedicine for medical abortion in the public sector to increase access to safe abortions in South Africa.

## Supplementary Information

Supplementary Material 1.

## Data Availability

All qualitative data were obtained from interviews with study participants. The data is not available publicly or upon request to protect study participants’ privacy.

## Abbreviations

COREQ: The Consolidated Criteria for Reporting Qualitative Research
COVID-19: Coronavirus disease 2019
HIV: Human immunodeficiency virus
ICT: Information and communication technology
OECD: The Organisation for Economic Co-operation and Development
RCT: Randomised controlled trial
WHO: World Health Organization
UK: United Kingdom
US: United States

## References

[1] Owolabi OO, Biddlecom A, Whitehead HS. Health systems’ capacity to provide post-abortion care: a multicountry analysis using signal functions. Lancet Glob Health. 2019;7(1):e110-8. 30503402 10.1016/S2214-109X(18)30404-2 PMC6478445

[2] Kapp N, Lohr PA. Modern methods to induce abortion: safety, efficacy and choice. Best Pract Res Clin Obstet Gynaecol. 2020;63:37–44. 32029379 10.1016/j.bpobgyn.2019.11.008

[3] World Health Organization. Abortion care guideline. Geneva: World Health Organization. 2022. Available from: https://www.who.int/publications-detail-redirect/9789240039483. Accessed 28 Dec 2024.

[4] Craig A, Beek K, Godinho M, Ansari S, Jonnagaddala J, Linhart C et al. Digital health and universal health coverage: opportunities and policy considerations for Pacific Island health authorities . New Delhi: World Health Organization Regional Office for South-East Asia; 2022. Available from: https://apps.who.int/iris/handle/10665/361160. Accessed 28 Dec 2024.

[5] Cleeve A, Lavelanet A, Gemzell-Danielsson K, Endler M. The use of telemedicine services for medical abortion. Cochrane Database Syst Rev. 2025;6(6):CD013764. 40464275 10.1002/14651858.CD013764.pub2 PMC12135146

[6] Hyland P, Raymond EG, Chong E. A direct-to-patient telemedicine abortion service in Australia: retrospective analysis of the first 18 months. Aust N Z J Obstet Gynaecol. 2018;58(3):335–40. 29603139 10.1111/ajo.12800

[7] Seymour JW, Melville C, Thompson TA, Grossman D. Effectiveness and safety of a direct-to-patient telehealth service providing medication abortion targeted at rural and remote populations: Cross-sectional findings from Marie Stopes Australia. Contraception. 2022;115:67–8. 35753405 10.1016/j.contraception.2022.06.010

[8] Peña M, Figueroa Flores K, Muñoz Ponce M, Facio Serafín D, Camarillo Zavala AM, Ruiz Cruz C, et al. Telemedicine for medical abortion service provision in Mexico: a safety, feasibility, and acceptability study. Contraception. 2022;114:67–73. 35753406 10.1016/j.contraception.2022.06.009

[9] Aiken A, Lohr P, Lord J, Ghosh N, Starling J. Effectiveness, safety and acceptability of no-test medical abortion (termination of pregnancy) provided via telemedicine: a national cohort study. BJOG Int J Obstet Gynaecol. 2021;128(9):1464–74. 10.1111/1471-0528.16668 PMC8360126 33605016

[10] Chong E, Shochet T, Raymond E, Platais I, Anger HA, Raidoo S, et al. Expansion of a direct-to-patient telemedicine abortion service in the United States and experience during the COVID-19 pandemic. Contraception. 2021;104(1):43–8. 33781762 10.1016/j.contraception.2021.03.019 PMC9748604

[11] Upadhyay UD, Raymond EG, Koenig LR, Coplon L, Gold M, Kaneshiro B, et al. Outcomes and safety of history-based screening for medication abortion: a retrospective multicenter cohort study. JAMA Intern Med. 2022;182(5):482–91. 35311911 10.1001/jamainternmed.2022.0217 PMC8938895

[12] Piay-Fernández N, Stenbacka E, Jaramillo MC, Guerrero G, Solano Rodríguez AA, Montenegro P, et al. Implementing medical abortion through telemedicine in Colombia: a qualitative study. Sex Reprod Health Matters. 2023;31(4):2236780. 37565788 10.1080/26410397.2023.2236780 PMC10424593

[13] Republic of South Africa. Choice on Termination of Pregnancy Act No. 92 of 1996. Government Gazette. 1996;377:17602. 11656779

[14] Kaswa R, Yogeswaran P. Abortion reforms in South Africa: an overview of the choice on termination of pregnancy act. South Afr Fam Pract. 2020;62(1):e1–5. 10.4102/safp.v62i1.5240 PMC8378188 33314950

[15] Favier M, Greenberg JMS, Stevens M. Safe abortion in South Africa: ‘We have wonderful laws but we don’t have people to implement those laws’. Int J Gynaecol Obstet Off Organ Int Fed Gynaecol Obstet. 2018;143(Suppl 4):38–44. 10.1002/ijgo.12676 30374986

[16] Gerdts C, DePiñeres T, Hajri S, Harries J, Hossain A, Puri M, et al. Denial of abortion in legal settings. J Fam Plann Reprod Health Care. 2015;41(3):161–3. 25511805 10.1136/jfprhc-2014-100999 PMC4501171

[17] Amnesty International. Barriers to safe and legal abortion in South Africa. Amnesty International Publications. 2017. Report No.: AFR53/5423/2017. Available from: https://www.amnesty.org/en/documents/afr53/5423/2017/en/. Accessed 28 Dec 2024.

[18] Harries J, Daskilewicz K, Bessenaar T, Gerdts C. Understanding abortion seeking care outside of formal health care settings in Cape Town, South Africa: a qualitative study. Reprod Health. 2021;18(1):190. 34556120 10.1186/s12978-021-01243-3 PMC8460179

[19] Dhlamini M. Pills and phone calls: How COVID restrictions forced us to conduct abortions telephonically. Bhekisisa. 2020. Available from: https://bhekisisa.org/article/2020-09-29-pills-and-phone-calls-how-covid-restrictions-forced-us-to-conduct-abortions-telephonically/. Accessed 10 Jan 2023.

[20] Marie Stopes South Africa. Safe abortion services and contraceptives. 2025. Available from: https://www.mariestopes.org.za/. Accessed 10 Aug 2025.

[21] Abortion Support South Africa. I need an abortion – safe abortion with pills at home. 2025. Available from: https://abortionsupport.co.za/. Accessed 10 Aug 2025.

[22] Rensburg R. New healthcare plan promises to overhaul South Africa’s massively skewed system. The Conversation. 2018. Available from: http://theconversation.com/new-healthcare-plan-promises-to-overhaul-south-africas-massively-skewed-system-99404. Accessed 28 Dec 2024.

[23] Endler M, Petro G, Gemzell Danielsson K, Grossman D, Gomperts R, Weinryb M, et al. A telemedicine model for abortion in South Africa: a randomised, controlled, non-inferiority trial. Lancet Lond Engl. 2022;400(10353):670–9. 10.1016/S0140-6736(22)01474-X 36030811

[24] Somefun OD, Constant D, Endler M. The acceptability of implementing telemedicine for early medical abortion in South Africa: a substudy to a randomized controlled trial. SSM - Qual Res Health. 2023;3:100241.

[25] Rapport F, Clay-Williams R, Churruca K, Shih P, Hogden A, Braithwaite J. The struggle of translating science into action: foundational concepts of implementation science. J Eval Clin Pract. 2018;24(1):117–26. 28371050 10.1111/jep.12741 PMC5901403

[26] Godfrey EM, Fiastro AE, Jacob-Files EA, Coeytaux FM, Wells ES, Ruben MR, et al. Factors associated with successful implementation of telehealth abortion in 4 United States clinical practice settings. Contraception. 2021;104(1):82–91. 33932401 10.1016/j.contraception.2021.04.021 PMC8542461

[27] Tong A, Sainsbury P, Craig J. Consolidated criteria for reporting qualitative research (COREQ): a 32-item checklist for interviews and focus groups. Int J Qual Health Care. 2007;19(6):349–57. 17872937 10.1093/intqhc/mzm042

[28] Tanahashi T. Health service coverage and its evaluation. Bull World Health Organ. 1978;56(2):295–303. 96953 PMC2395571

[29] Baker U, Peterson S, Marchant T, Mbaruku G, Temu S, Manzi F, et al. Identifying implementation bottlenecks for maternal and newborn health interventions in rural districts of the United Republic of Tanzania. Bull World Health Organ. 2015;93(6):380–9. 26240459 10.2471/BLT.14.141879 PMC4450702

[30] Dedoose LLC. 2023. Available from: https://www.dedoose.com. Accessed 28 Dec 2024.

[31] Vears DF, Gillam L. Inductive content analysis: a guide for beginning qualitative researchers. Focus Health Prof Educ Multi-Prof J. 2022;23(1):111–27.

[32] Killinger K, Günther S, Gomperts R, Atay H, Endler M. Why women choose abortion through telemedicine outside the formal health sector in Germany: a mixed-methods study. BMJ Sex Reprod Health. 2022;48(e1):e6–12. 33229399 10.1136/bmjsrh-2020-200789

[33] Sorhaindo AM, Lavelanet AF. Why does abortion stigma matter? A scoping review and hybrid analysis of qualitative evidence illustrating the role of stigma in the quality of abortion care. Soc Sci Med. 2022;311:115271. 36152401 10.1016/j.socscimed.2022.115271 PMC9577010

[34] Peltzer K, Parker W, Mabaso M, Makonko E, Zuma K, Ramlagan S. Impact of national HIV and AIDS communication campaigns in South Africa to reduce HIV risk behaviour. Sci World J. 2012;2012:e384608. 10.1100/2012/384608 PMC3504395 23213285

[35] Jack CL, Mars M. Ethical considerations of mobile phone use by patients in KwaZulu-Natal: Obstacles for mHealth? Afr J Prim Health Care Fam Med. 2014;6(1):7. 10.4102/phcfm.v6i1.607 PMC4502882 26245406

[36] Sharma AE, Lisker S, Fields JD, Aulakh V, Figoni K, Jones ME, et al. Language-specific challenges and solutions for equitable telemedicine implementation in the primary care safety net during COVID-19. J Gen Intern Med. 2023;38(14):3123–33. 37653210 10.1007/s11606-023-08304-2 PMC10651814

[37] Ayo-Farai O, Ogundairo O, Maduka CP, Okongwu CC, Babarinde AO, Sodamade OT. Telemedicine in health care: a review of progress and challenges in Africa. Matrix Sci Pharma. 2023;7(4):124.

[38] Mash R, Christian C, Chigwanda RV. Alternative mechanisms for delivery of medication in South Africa: a scoping review. South Afr Fam Pract. 2021;63(3):a5274. 10.4102/safp.v63i1.5274 PMC8424755 34476963

[39] Dodoo JE, Al-Samarraie H, Alzahrani AI. Telemedicine use in Sub-Saharan Africa: barriers and policy recommendations for Covid-19 and beyond. Int J Med Inf. 2021;151:104467. 10.1016/j.ijmedinf.2021.104467 PMC9761083 33915421

[40] Eze ND, Mateus C, Hashiguchi TCO. Telemedicine in the OECD: an umbrella review of clinical and cost-effectiveness, patient experience and implementation. PLoS ONE. 2020;15(8):e0237585. 32790752 10.1371/journal.pone.0237585 PMC7425977

[41] Sibuyi IN, de la Harpe R, Nyasulu P. A stakeholder-centered mHealth implementation inquiry within the digital health innovation ecosystem in South Africa: MomConnect as a demonstration case. JMIR MHealth UHealth. 2022;10(6):e18188 35708756 10.2196/18188 PMC9247812

[42] Constant D, Harries J, Moodley J, Myer L. Accuracy of gestational age estimation from last menstrual period among women seeking abortion in South Africa, with a view to task sharing: a mixed methods study. Reprod Health. 2017;14(1):100. 28830534 10.1186/s12978-017-0365-7 PMC5568056

[43] Harries J, Constant D. Providing safe abortion services: experiences and perspectives of providers in South Africa. Best Pract Res Clin Obstet Gynaecol. 2020;62:79–89. 31279763 10.1016/j.bpobgyn.2019.05.005

