# Supplementary material for: Implementing telemedicine for medical abortion within the public health system: a qualitative study on implementation bottlenecks and solutions in South Africa
Source: BMC Public Health. 2025 Oct 9;25:3421. doi: 10.1186/s12889-025-24690-0 (PMC12512679; doi:10.1186/s12889-025-24690-0)
Supplement: Supplementary file 1 — Supplementary Material 1. [file 12889_2025_24690_MOESM1_ESM.docx]

### **Interview Guide**

Questions:

What are your experiences with medical abortion through telemedicine in South Africa?

What do you think about telemedicine as a service delivery model for medical abortion?

This service isn’t currently available through the public sector. What do you think are some factors currently preventing this?

What needs to change for medical abortion through telemedicine to work in the public sector?

How ready/willing is the public healthcare system to take this on?

What does a successful model of medical abortion through telemedicine in South Africa look like to you?
